# Comparative transcriptomics between *Drosophila mojavensis* and *D. arizonae* reveals transgressive gene expression and underexpression of spermatogenesis-related genes in hybrid testes

**DOI:** 10.1038/s41598-021-89366-2

**Published:** 2021-05-10

**Authors:** Cecilia A. Banho, Vincent Mérel, Thiago Y. K. Oliveira, Claudia M. A. Carareto, Cristina Vieira

**Affiliations:** 1 Department of Biology, UNESP - São Paulo State University, São José do Rio Preto, São Paulo State (SP) Brazil; 2 Laboratoire de Biométrie et Biologie Evolutive, CNRS, UMR 5558, Université Claude Bernard Lyon 1, University of Lyon, 69622 Villeurbanne, France; 3 Laboratory of Molecular Immunology, The Rockefeller University, New York, NY USA

**Keywords:** Evolution, Genetics

## Abstract

Interspecific hybridization is a stressful condition that can lead to sterility and/or inviability through improper gene regulation in *Drosophila* species with a high divergence time. However, the extent of these abnormalities in hybrids of recently diverging species is not well known. Some studies have shown that in *Drosophila*, the mechanisms of postzygotic isolation may evolve more rapidly in males than in females and that the degree of viability and sterility is associated with the genetic distance between species. Here, we used transcriptomic comparisons between two *Drosophila mojavensis* subspecies and *D. arizonae* (*repleta* group, *Drosophila*) and identified greater differential gene expression in testes than in ovaries. We tested the hypothesis that the severity of the interspecies hybrid phenotype is associated with the degree of gene misregulation. We showed limited gene misregulation in fertile females and an increase in the amount of misregulation in males with more severe sterile phenotypes (motile vs. amotile sperm). In addition, for these hybrids, we identified candidate genes that were mostly associated with spermatogenesis dysfunction.

## Introduction

Speciation is a complex process resulting from the divergence of two populations from an ancestral lineage by reproductive barriers capable of preventing gene flow^1,2^. Among these barriers, postzygotic isolation mechanisms contribute to hybrid incompatibility, and their consequences can be observed by the presence of two main traits, hybrid sterility and/or hybrid inviability, which can evolve at different rates. Overall, hybrid sterility evolves faster than hybrid inviability, mainly when considering the different sexes, since these barriers evolve faster in males than in females^3^. Indeed, several studies considering intraspecific and interspecific hybridization have shown that the germline is primarily affected, and sterility is often detected^4–10^. Although the process of speciation has been widely studied, the causes of postzygotic incompatibility in hybrids are not fully understood. According to the model of Dobzhansky–Muller^11,12^, fixed mutations in genetically isolated populations can result in deleterious epistatic interactions, disrupting regulatory networks and leading to serious consequences in hybrids^2,13–15^. In addition, according to Haldane’s rule^16^, sterility in hybrids is more likely to affect the heterogametic sex, and the degree and extent of these genetic incompatibilities are related to the time of divergence and are likely the result of divergent regulatory sequences^13,15,17,18^.

Most of the hybrid incompatibility genes identified so far seem to have species-specific effects leading to hybrid incompatibility, indicating that this is a complex polygenic trait that has different epistatic effects, depending on the pair of species, which is likely related to the rapid accumulation of genetic changes over time. Studies of recently diverged species presenting different phenotypes regarding postzygotic isolation mechanisms can help to clarify early disruptions in gene regulation and expression that may influence the speciation process. *Drosophila mojavensis* and *D. arizonae* are appropriate for such a study. They are sibling species with divergence time estimates from 0.66–0.99 mya^19^ to 4.2 ± 0.99 mya^20^, depending on the study. The most recent data, based on more than 5000 genes, indicate ~ 1.5 mya^21^ of divergence between the two species. This pair of species is widely used in speciation studies due to their ability to produce hybrids in the laboratory; however, introgression has not yet been found in nature despite the favourable ecological conditions for hybridization, mainly between sympatric populations^22^. *D. mojavensis* is endemic to the southwestern United States and northwestern Mexico. It is composed of four ecologically distinct subspecies, which are distributed in four different geographic regions, and each subspecies uses a specific host cactus as a feeding and breeding site, with no evidence of recent gene flow, constituting well-structured populations^19,23–27^. In crosses between *D. mojavensis* subspecies and *D. arizonae*, hybrids can be produced in both directions. Nevertheless, they present incomplete and asymmetric postzygotic isolation, since in crosses with *D. mojavensis* females, the sterility of the male hybrids is variable, whereas in crosses with *D. arizonae* females, the male hybrids are always sterile because they do not have motile sperm^10,28,29^.

With this in mind, we sought to assess the magnitude of differential gene expression (DEG) in male and female offspring from *D. arizonae *×* D. mojavensis* crosses and its association with hybrid phenotypes. We showed that in fertile hybrid females, very few DEGs were identified. However, in male hybrids, the degree of deregulation of gene expression was related to the severity of the sterile phenotype because males without motile spermatozoa had several DEGs, with a bias for underexpression, and the functions of these genes were related to spermatogenesis. By contrast, in sterile hybrids with motile spermatozoa, the degree of deregulation was lower and had a bias toward overexpression, and the gene functions were not directly related to spermatogenesis.

## Results

**Figure 1:**
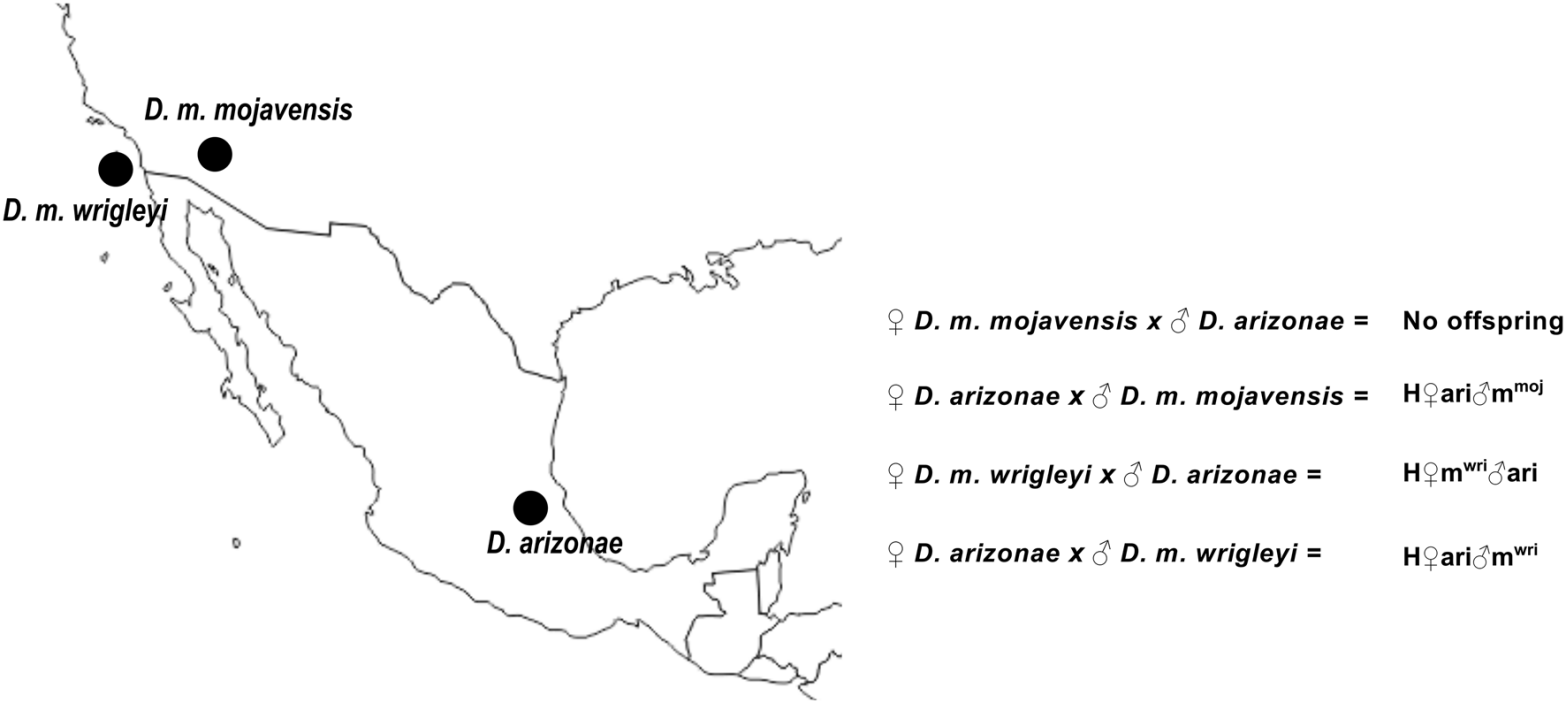
Crosses performed between *D. arizonae* and *D. mojavensis* subspecies and differential gene expression between parental lines and hybrids. Geographic distribution of *D. m. wrigleyi, D. m. mojavensis* and *D. arizonae* lines used in this study and their respective cross directions with offspring identifications (http://www.d-maps.com/carte.php?num_car=1404&lang=en). H♀m^wri^♂ari and H♀ari♂m^wri^ are offspring from crosses between *D. arizonae* and *D. m. wrigleyi*, and H♀ari♂m^moj^ are hybrids from crosses between *D. arizonae* and *D. m. mojavensis*.

The transcriptomes of the ovaries and testes of three parental allopatric strains (*D. m. mojavensis*, *D. m. wrigleyi* and *D. arizonae*) and their reciprocal hybrids (Fig. 1) were sequenced. We were able to recover 11,654 coding genes from *D. mojavensis* r1.04 transcripts. Of those, 9321 (80%) genes were expressed in *D. arizonae, D. m. mojavensis* and H♀ari♂m^moj^ ovaries, and 9700 (83.3%) were expressed in *D. arizonae*, *D. m. wrigleyi*, H♀m^wri^♂ari and H♀ari♂m^wri^ female gonads. In testes, we found 11,146 (95.6%) genes expressed in *D. arizonae, D. m. mojavensis* and H♀ari♂m^moj^ and 11,223 (96.3%) expressed in *D. arizonae*, *D. m. wrigleyi*, H♀m^wri^♂ari and H♀ari♂m^wri^. The read alignment rate ranged from 81.7 to 86% in ovaries and from 76.4 to 80.8% in testes (Supplementary Table S1). A similar alignment rate for *D. arizonae*, *D. mojavensis* subspecies and their reciprocal hybrids indicated that the genome of the *D. mojavensis* r1.04 reference can be used in analyses involving *D. arizonae* and hybrids. Additionally, according to Lopez-Maestre et al.^30^, the average nucleotide divergence between *D. arizonae* and *D. mojavensis* is very low, less than 2%; thus, the sequence alignments of parental species and their hybrids with the r1.04 genome of *D. mojavensis* should not affect mapping efficiency. Principal component analysis (PCA) was performed to verify the variance of the biological replicates. Within ovaries and testes, the replicates were grouped together (see Supplementary Fig. S1), indicating low variance between replicates.

### Differential gene expression in *D. arizonae* and *D. mojavensis* gonads

**Figure 2:**
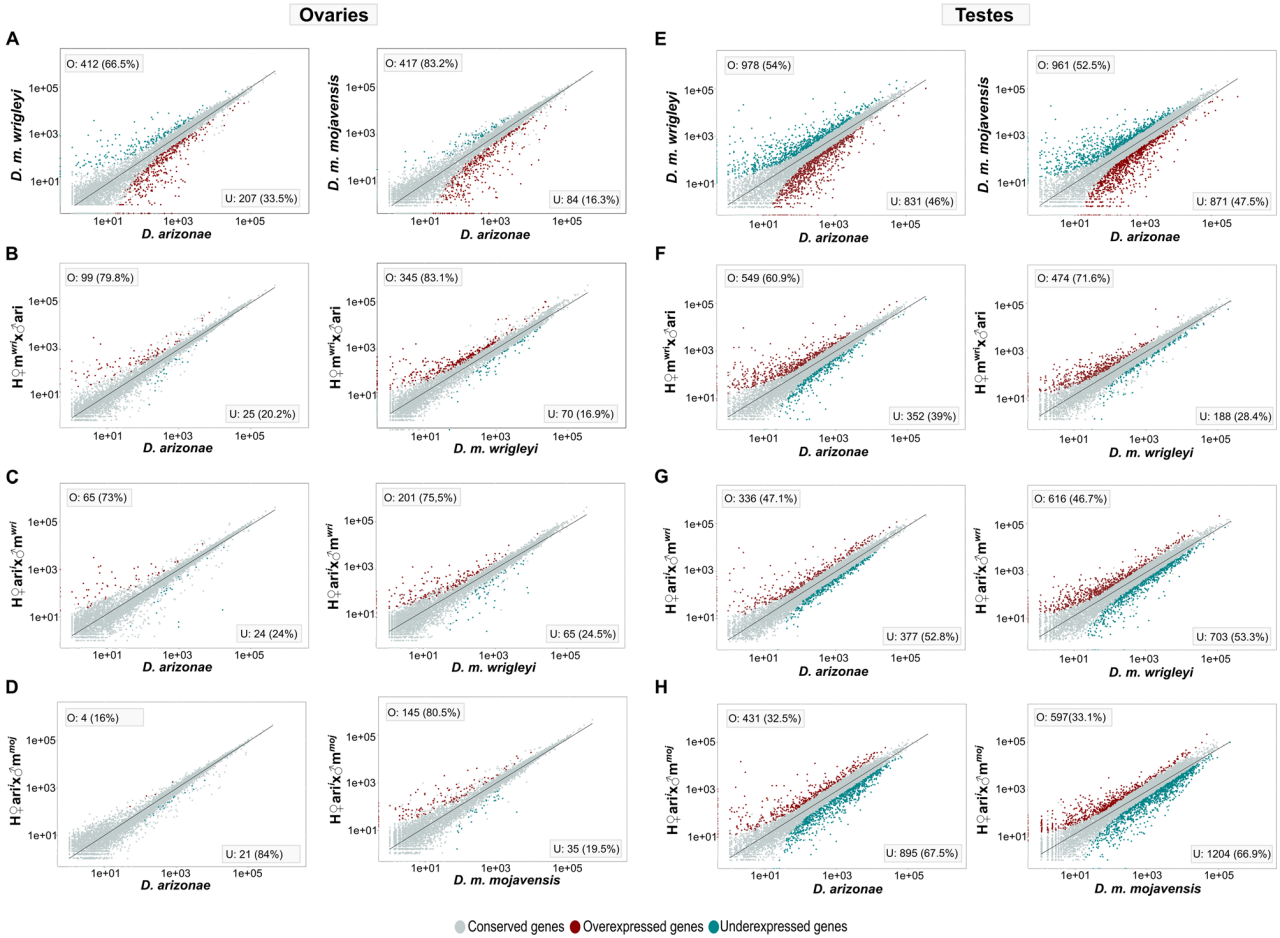
Differential gene expression between *D. arizonae* and *D. mojavensis* subspecies and between hybrids and their respective parental lines. Scatter plots representing differential gene expression in ovaries (left) and testes (right) between parental lines, as well as between hybrids and their respective parental lines. Genes were considered differentially expressed when they presented twofold differences and FDR-corrected *p*-values < 0.01. Red dots = overexpressed genes; blue dots = underexpressed genes; grey dots = not differentially expressed. (**A**) Differential expression in ovaries of *D. m. mojavensis* × *D. arizonae* and *D. m. wrigleyi* × *D. arizonae* hybrids. (**B**) Differential expression in ovaries of H♀m^wri^♂ari × *D. m. wrigleyi* hybrids and *D. arizonae*. (**C**) Differential expression in ovaries of H♀ari♂m^wri^ × *D. m. wrigleyi* hybrids and *D. arizonae*. (**D**) Differential expression in ovaries of H♀ari♂m^moj^ × *D. m. mojavensis* hybrids and *D. arizonae*. (**E**) Differential expression in testes of *D. m. mojavensis* × *D. arizonae* and *D. m. wrigleyi* × *D. arizonae* hybrids. (**F**) Differential expression in testes of H♀m^wri^♂ari × *D. m. wrigley*i hybrids and *D. arizonae*. (**G**) Differential expression in testes of H♀ari♂m^wri^ × *D. m. wrigleyi* hybrids and *D. arizonae*. (**H**) Differential expression in testes of H♀ari♂m^moj^ × *D. m. mojavensis* hybrids and *D. arizonae*. The scatter plot was generated using the ggplot2 package (version 3.3.3) in R^79^. O: overexpressed genes; U: underexpressed genes.

**Figure 3:**
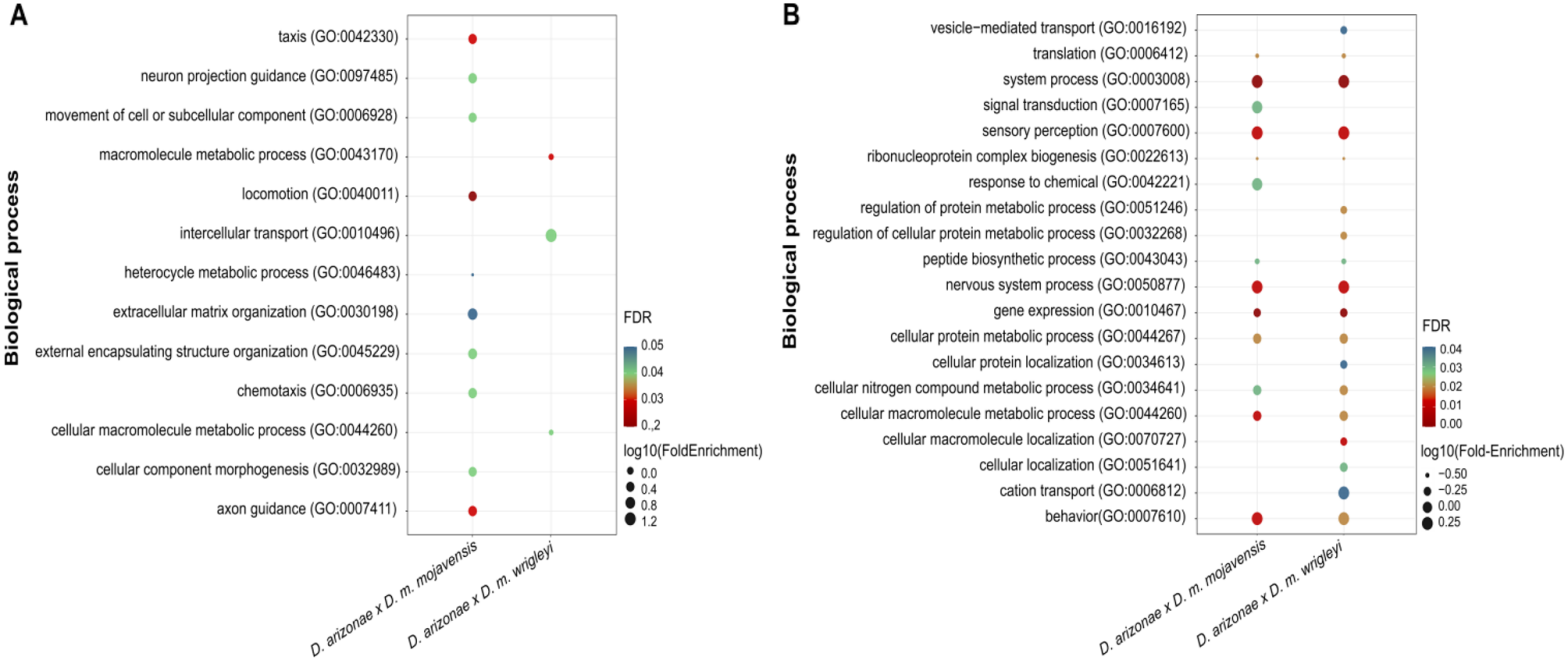
GO term enrichment analysis of DEGs between *D. arizonae* and *D. mojavensis* subspecies. Dot plot representing GO enrichment for the biological process category for DEGs in (**A**) ovaries and (**B**) testes between *D. arizonae*- *D. m. mojavensis* and *D. arizonae- D. m. wrigleyi* (FDR < 0.05). The dot plot was generated using the ggplot2 package (version 3.3.3) in R^79^.

We compared the transcriptomes of *D. arizonae* and *D. m. mojavensis* and those of *D. arizonae* and *D. m. wrigleyi* and identified 501 (5.3%) and 619 (6.3%) DEGs in ovaries, respectively, and the majority of the genes were overexpressed in *D. arizonae* (X^2^ = 220, *p* = 2.2e−16; X^2^ = 67.231, *p* = 2.415e−16, respectively) (Fig. 2A, Supplementary Table S6). In testes, we observed that 16% of genes were differentially expressed (Fig. 2E) when comparing *D. arizonae* with both *D. mojavensis* subspecies, and there was a higher proportion of overexpressed genes in both comparisons (X^2^ = 4.34, *p* = 0.03759; X^2^ = 11.783, *p* = 0.0005976) (Fig. 2E, see Supplementary Table S6). Gene enrichment analyses between parental lines showed that in ovaries, the DEGs were mainly enriched for nervous system, metabolism, chemotaxis, and extracellular matrix organization, among others. In testes, DEGs were also enriched for metabolic function, sensory perception, nervous system, gene expression, and behaviour, among other functions (Fig. 3, Supplementary Tables S2 and S3 and Supplementary Fig. S2 for cellular component and molecular function enrichment). Among the DEGs with functions in metabolic processes, those involved in processes of oxidation–reduction and metabolism of carbohydrates and lipids can be highlighted (see Supplementary Tables S2, S3, S4 and S5).

### Gene expression in hybrid female gonads is very similar to that in the parental lines

We compared the patterns of gene expression between hybrids and their parental lines. In hybrids between *D. m. wrigleyi* and *D. arizonae*, the percentage of DEGs between the hybrids and the parental lines ranged from 1.27 to 4.2% (Supplementary Table S7). For all the comparisons, the proportion of overexpressed genes was significantly higher than that of underexpressed genes and was always higher in female hybrids from *D. arizonae-D. m. wrigleyi* crosses (Fig. 2A–C, see Supplementary Table S7). In hybrids between *D. m. mojavensis* and *D. arizonae*, the percentage of DEGs between the hybrids and the parental lines was 0.26 and 1.93% when compared with *D. arizonae* and *D. m. mojavensis,* respectively (Supplementary Table S7). The proportion of overexpressed versus underexpressed genes was significantly different and dependent on the cross (Supplementary Table S7). When comparing H♀ari♂m^moj^ with *D. arizonae,* there was a higher proportion of underexpressed genes than overexpressed genes (X^2^ = 10.24, *p* = 0.001374), contrary to the comparison between H♀ari♂m^moj^ and *D. m. mojavensis,* for which we identified a higher proportion of overexpressed genes in the hybrids (X^2^ = 66.006, *p* = 4.497e−16) (Fig. 2D).

**Figure 4:**
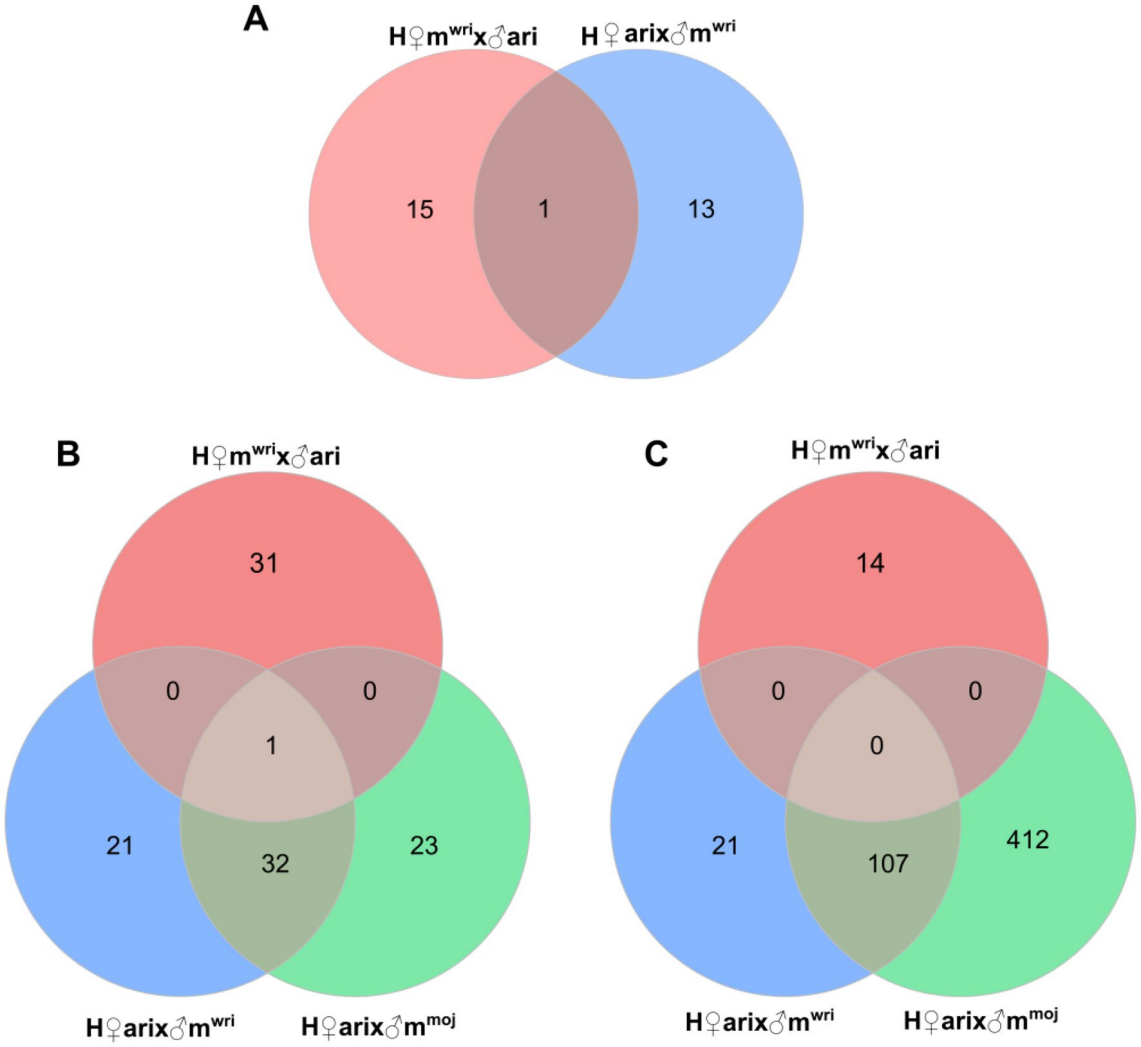
Shared DEGs between hybrids. (**A**) Number of shared overexpressed genes between H♀m^wri^♂ari and H♀ari♂m^wri^ females. (**B**) Number of shared overexpressed genes between H♀m^wri^♂ari, H♀ari♂m^wri^ and H♀ari♂m^moj^ males. (**C**) Number of shared underexpressed genes between H♀m^wri^♂ari, H♀ari♂m^wri^ and H♀ari♂m^moj^ males. A Venn diagram was generated using the VennDiagram package (version 1.6.20) in R^79^.

By filtering the genes that were over- or underexpressed relative to both parental lines for all crosses, we greatly reduced our set of DEGs. From the DEGs in H♀m^wri^♂ari ovaries, 16 genes were overexpressed in relation to both parental lines (Supplementary Table S8); however, no significant GO enrichment was found. Moreover, no underexpressed genes were found. H♀ari♂m^wri^ ovaries showed 13 overexpressed genes (Supplementary Table S9) and only one underexpressed gene (FBgn0135298, with unknown function) when compared with both parental lines. Additionally, H♀m^wri^♂ari and H♀ari♂m^wri^ ovaries showed only one shared overexpressed gene, FBgn0145754 (unknown function) (Fig. 4A). In H♀ari♂m^moj^ ovaries, no overexpressed genes were found, while only three genes were underexpressed. These genes included FBgn014602, which corresponds to *alpha-Est5* and has carboxylic ester hydrolase activity; FBgn028050, corresponding to the *Maverick* gene, which has a role in signalling pathways, the regulation of neuromuscular junctions, dendrite development and imaginal disc-derived wing size; and FBgn0146651, orthologous to CG11854 in *D. melanogaster,* which is involved in reproduction, more specifically in courtship behaviour^31,32^.

### Gene expression in the hybrid male germline is transgressive when compared to the parental lines

In comparison to ovaries, hybrid testes always presented a larger number of DEGs in relation to parental lines (Fig. 2E, Supplementary Table S7). In H♀m^wri^♂ari testes, we found that 8% and 5.8% of genes were differentially expressed compared with *D. arizonae* and *D. m. wrigleyi*, respectively, which displayed a significant bias for overexpression (X^2^ = 42.637, *p* = 6.59e−11; X^2^ = 122.7, *p* = 2.2e−16, Fig. 2F, Supplementary Table S7). In hybrid testes from the reciprocal cross (H♀ari♂m^wri^), we found that 6.3% and 11.75% of genes were differentially expressed compared with *D. arizonae* and *D. m. wrigleyi* (Fig. 2G, Supplementary Table S7). Unlike H♀m^wri^♂ari testes, no bias towards over- or underexpression was observed in the comparison with *D. arizonae* (X^2^ = 2.244, *p* = 0.1341) (see Supplementary Table S7). In testes from H♀ari♂m^moj^, we observed that 11.9% and 16% of genes were differentially expressed compared with *D. arizonae* and *D. m. mojavensis*, respectively, with a significant proportion of underexpressed genes, 67.5% and 66.9%, respectively (X^2^ = 162.24, *p* = 2.2e−16, X^2^ = 203.91, *p* = 2.2e−16) (Fig. 2H, Supplementary Table S7).

Regarding the DEGs in H♀m^wri^♂ari testes, 32 were overexpressed and 14 were underexpressed when compared with both parental species, while in H♀ari♂m^wri^ testes, 56 and 128 genes were over- and underexpressed, respectively, when compared with *D. arizonae* and *D. m. wrigleyi*. Similar to those of H♀ari♂m^wri^, testes of H♀ari♂m^moj^ displayed more underexpressed genes (519) than overexpressed genes (57) when compared to both parental species.

### Spermatogenesis-related functions of DEGs in testes

In the male gonads, the functions of differentially expressed genes differed depending on the direction of the cross. In H♀m^wri^♂ari testes, of the 46 DEGs found relative to both parental species, 39 had an orthologue in *D. melanogaster*. Most of the DEGs presented functional annotations associated with several functions, but no significant enrichment for GO terms was observed (see Supplementary Tables S10 and S11). On the other hand, in H♀ari♂m^wri^ testes, 183 genes were differentially expressed in both species, and 131 *D. melanogaster* orthologues were recovered. Functional annotation of these genes showed that several of them were related to processes such as reproduction and cellular division, including spermatogenesis, cilium movement, microtubule-based movement and nucleation, sperm motility, chromosome segregation and mitotic spindle elongation, as well as other functions related to several metabolic processes (*Cyp6a9/Cyp6a21, Ugt50B3, Gpo2*, CG7140, *P5CDh2*, *inaE*), transcription (*zen2, hb, Hr3, vnd, ap*) and sensory perception (*Obp8a, Or98a*) (see Supplementary Tables S12 and S13). In H♀ari♂m^moj^ testes, 576 genes were identified as differentially expressed, and 440 had a *D. melanogaster* orthologue. In these hybrids, most of the underexpressed genes were functionally annotated as having similar functions as those found in H♀ari♂m^wri^_,_ but it is important to emphasize some other functions, such as spermatid differentiation and development (Supplementary Tables S14 and S15).

**Figure 5:**
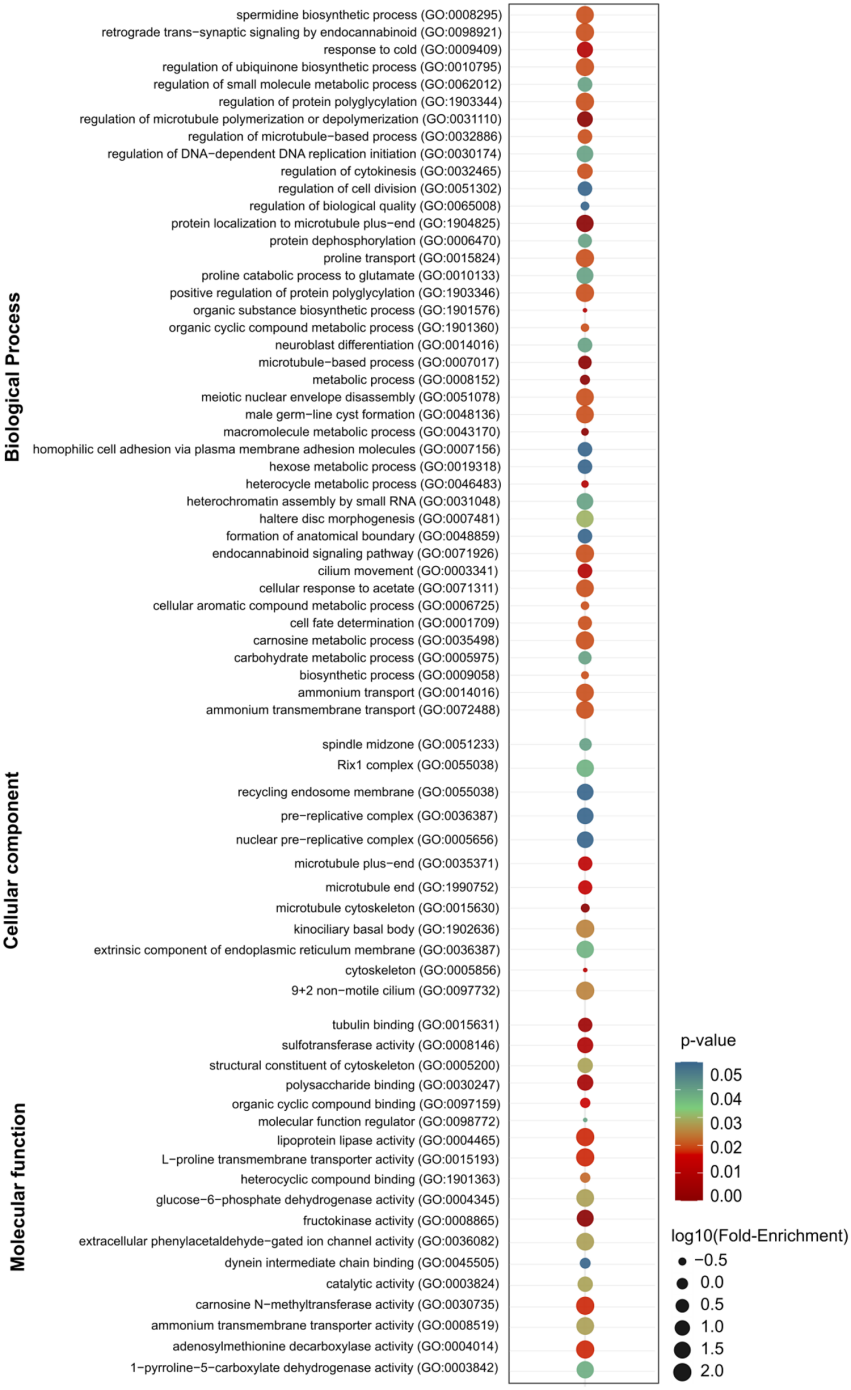
Functions of shared over- and underexpressed genes between H♀ari♂m^wri^ and H♀ari♂m^moj^ males. Dot plot representing GO enrichment for the biological process, cellular component and molecular function categories. The plot was generated using the ggplot2 package (version 3.3.3) in R^79^.

We searched for shared DEGs between hybrids and found that H♀m^wri^♂ari and H♀ari♂m^wri^ testes shared only one overexpressed gene (Fig. 4B). However, when comparing male gonad expression from crosses of H♀ari♂m^moj^ and H♀ari♂m^wri^, which have the same *D. arizonae* mother but two different *D. mojavensis* subspecies fathers, we identified 33 shared overexpressed genes (Fig. 4B, Supplementary Table S16) and 107 shared underexpressed genes (Fig. 4C, Supplementary Table S17). Interestingly, some of these genes exhibited GO term enrichment for spermatogenesis-related functions, such as regulation of cytokinesis and cell division, protein localization to the microtubule plus-end, microtubule-based process, male germline cyst formation and cilium movement, in addition to various metabolic processes (Fig. 5). Based solely on these reproductive genes, it is remarkable that some of them might have a direct role in reproduction, since they participate in male meiosis, male courtship, and mating behaviour, and play a role in gene silencing and pre-miRNA processing. Moreover, for many of these shared DEGs, the mutant phenotype has already been described for other *Drosophila* and corresponds to male sterility (Table 1)^33–49^.

**Table 1:** DEG in male hybrids gonads with reproductive functions.

*D. mojavensis* ID	D. *melanogaster* ID	Gene name	Biological process	Mutant phenotype	References	Expression		
						H♀m^wri^♂ari	H♀ari♂m^wri^	H♀ari♂m^moj^
FBgn0140112	FBgn0028858	*CG10839*	GO:0007018—microtubule-based movement	–	–	Not DF	Under	Under
FBgn0145039	FBgn0039812	*CG15548*	GO:0000022—mitotic spindle elongation	–	–	Not DF	Under	Under
FBgn0135509	FBgn0035581	*Dnah3*	GO:0007018—microtubule-based movement	Amotile sperm	^41^	Not DF	Under	Under
FBgn0140104	FBgn0028901	*CG18109*	GO:0007020—microtubule nucleation	–	–	Not DF	Under	Under
FBgn0147429	FBgn0063261	*CG31275*	GO:0007018—microtubule-based movement	–	–	Not DF	Under	Under
FBgn0142040	FBgn0027066	*Eb1*	GO:0000226: microtubule cytoskeleton organization	Defects in spindle elongation and orientation; reduction in astral microtubules	^44^	Not DF	Under	Under
FBgn0143126	FBgn0032225	*CG5022*	GO:0031032—actomyosin structure organization	–	–	Not DF	Not DF	Under
FBgn0137431	FBgn0283476	*Dhc16F*	GO:0001539—cilium or flagellum-dependent cell motility	Male sterility	^49^	Not DF	Not DF	Under
FBgn0136866	FBgn0039925	*Kif3C*	GO:0007018—microtubule-based movement	Roles in flagellar/ciliary motilities	^38,39^	Not DF	Not DF	Under
FBgn0140824	FBgn0031952	*cdc14*	GO:0071850—mitotic cell cycle arrest	–	–	Not DF	Under	Under
FBgn0140999	FBgn0262123	*l(2)41Ab*	GO:0070286—axonemal dynein complex assembly	–	–	Not DF	Under	Under
FBgn0146436	FBgn0038565	*CG7794*	GO:0007017—microtubule-based process	–	–	Not DF	Under	Under
FBgn0280294	FBgn0023090	*dtr*	GO:0060271—cilium morphogenesis	Male sterility	^49^	Not DF	Not DF	Under
FBgn0140392	FBgn0002673	*twe*	GO:0007140—male meiosis, GO:0007283—spermatogenesis	Male sterility. Absence of meiotic divisions in male germline and no motile sperm	^34,43^	Not DF	Under	Under
FBgn0144612	FBgn0267326	*Ntl*	GO:0030317—sperm motility	Male sterility. Amotile sperm and fail to be transferred to the seminal vesicle	^33^	Not DF	Under	Under
FBgn0281134	FBgn0001313	*kl2*	GO:0003341: cilium movement	Male sterility. Defects in sperm individualization	^48^	Not DF	Under	Not DF
FBgn0142949	FBgn0265512	*mlt*	GO:0007291—sperm individualization	Male sterility. Defects in sperm individualization	^37^	Not DF	Not DF	Under
FBgn0142705	FBgn0002865/ FBgn0004171	*Mst98Ca/ Mst98Cb*	GO:0007286—spermatid development	Structural proteins in the sperm tail	^45^	Not DF	Not DF	Under
FBgn0146037	FBgn0260942	*bond*	GO:0007112—male meiosis cytokinesis	Failure of cytokinesis in dividing spermatocytes	^46^	Not DF	Not DF	Under
FBgn0138561	FBgn0052529	*Hers*	GO:0006342: chromatin silencing	Affect the regulation process of cell proliferation/differentiation	^40^	Not DF	Over	Over
FBgn0141892	FBgn0010052	*Jhe*	GO:0060179—male mating behavior	Reduced courtship mutant males	^36,47^	Not DF	Not DF	Over
FBgn0140780	FBgn0086681/ FBgn0261349	*mst36F/ mst36Fb*	GO:0007018: microtubule-based movement	Reduction of male fertility	^35^	Not DF	Not DF	Under

Deregulated genes in male hybrid gonads presenting spermatogenesis-related functions and their mutant phenotype in other *Drosophila* species associated to them.

DF, Differentially expressed; Under, Underexpressed; Over, Overexpressed.

**Figure 6:**
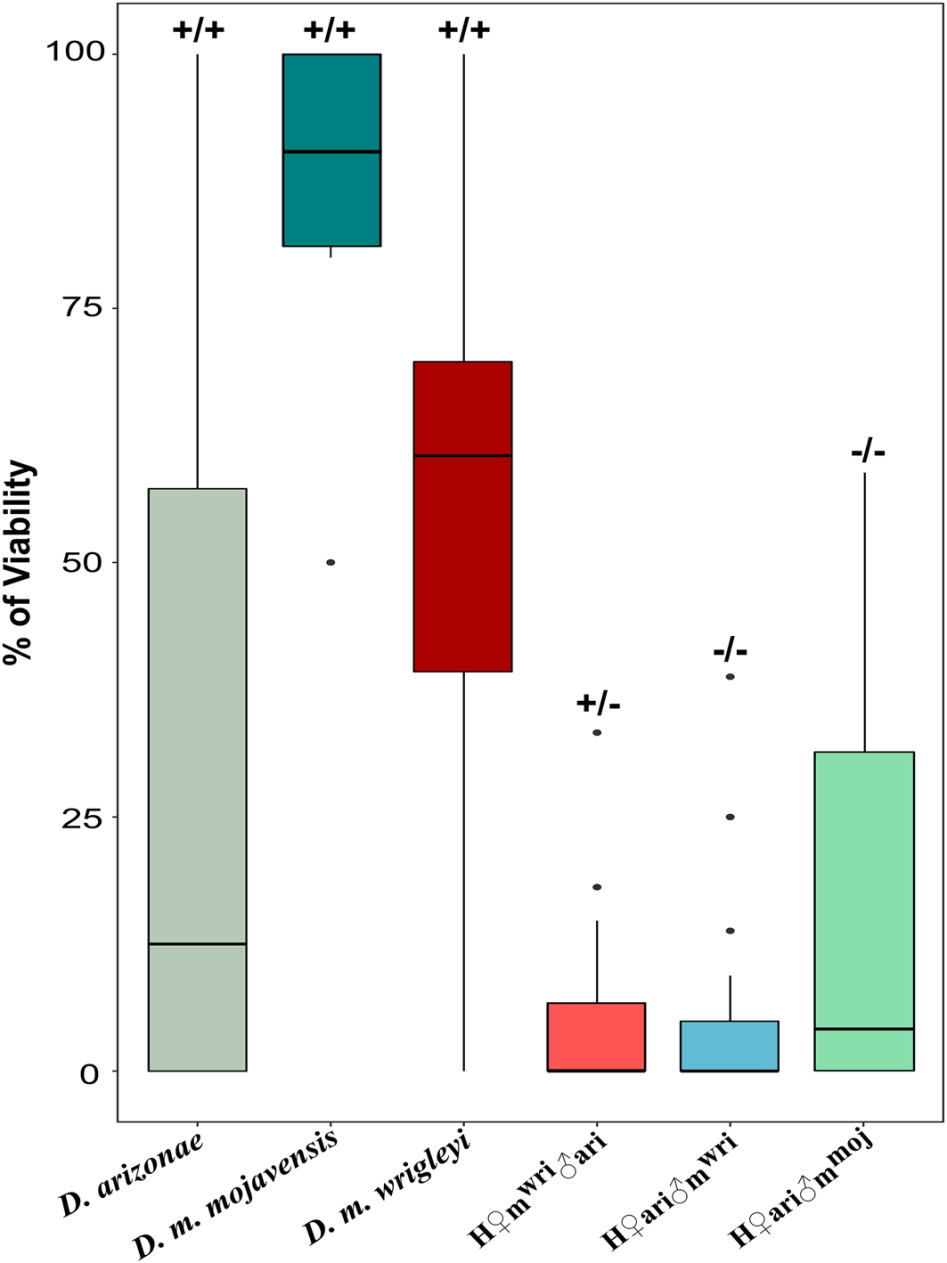
Average viability and fertility of intraspecific and interspecific offspring. Average viability (%) of *D. arizonae, D. m. wrigleyi* and *D. m. mojavensis* and their respective hybrids, H♀m^wri^♂ari, H♀ari♂m^wri^ and H♀ari♂m^moj^. Sperm motility and fertility are represented by—and + signals. +/+: motile sperm/fertile, +/−: motile sperm/sterile, −/− amotile sperm/sterile. The boxplot was generated using the ggplot2 package (version 3.3.3) in R^79^.

To better understand the results obtained for hybrid males, we examined some life-history traits, such as viability, sperm motility and fertility. Our analyses revealed that the viability of the hybrid was significantly reduced when compared to that of the parental lines (see Supplementary Table S18), since the average viability ranged from 3.85 to 14.2% in H♀ari♂m^moj^, H♀m^wri^♂ari and H♀ari♂m^wri^, while in the parental lines, it ranged from 29.58 to 86.8% (Fig. 6, Supplementary Table S18). Additionally, we found that all hybrid females were fertile. Regarding F_1_ males from the two types of crosses, we observed two phenotypes. H♀m^wri^♂ari males produced motile sperm but were sterile, since no offspring were produced in backcrosses and F_1_xF_1_ crosses. In contrast, H♀ari♂m^wri^ and H♀ari♂m^moj^ males produced amotile sperm and were also sterile (Fig. 6, Supplementary Table S18). These results suggest a link between the severity of the sterile phenotype and the number and functions of DEGs in male hybrids from crosses with *D. arizonae* mothers.

### Inheritance of gene expression

**Figure 7:**
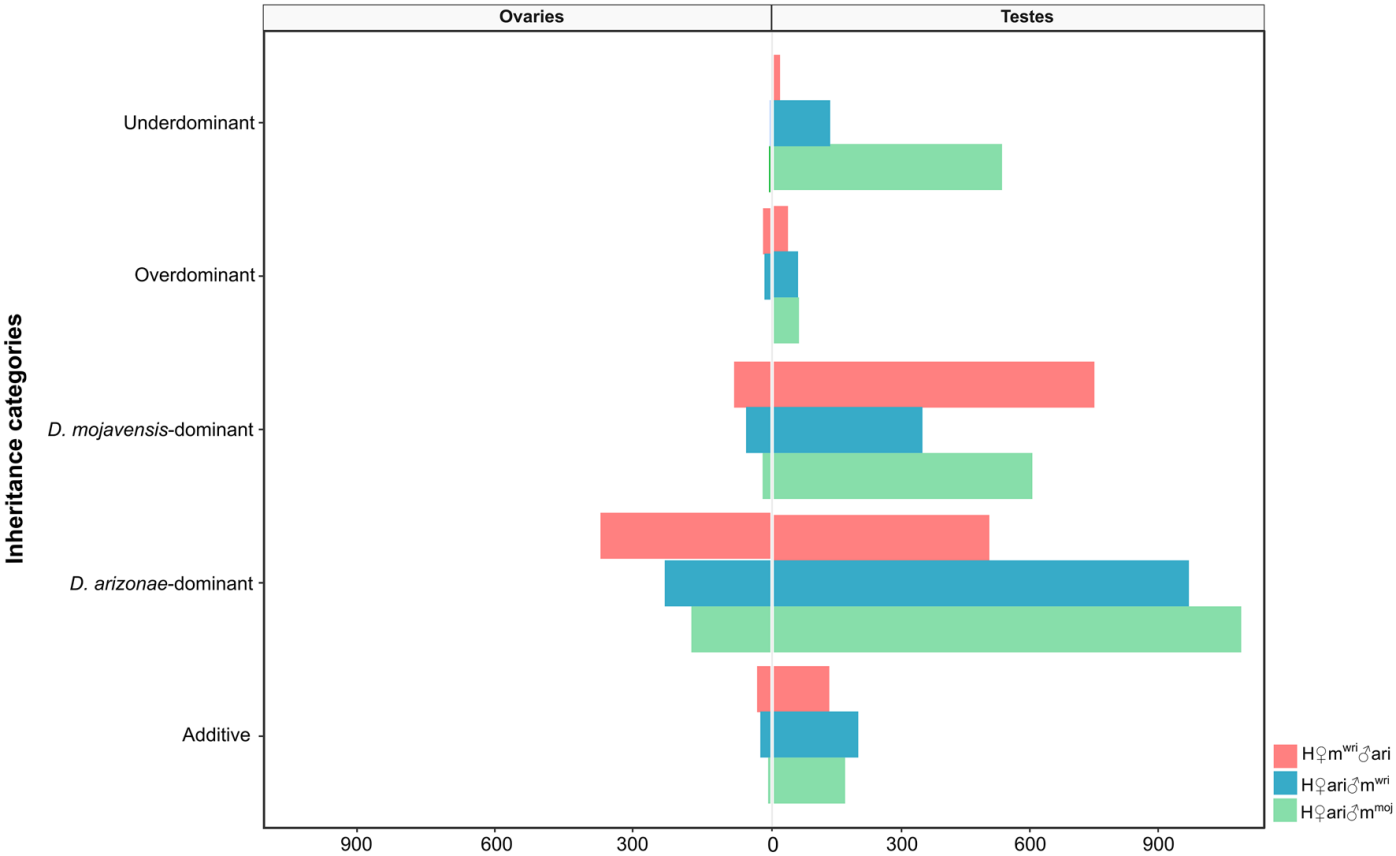
Inheritance of gene expression patterns in ovaries and testes of H♀m^wri^♂ari, H♀ari♂m^wri^ and H♀ari♂m^moj^. Gene expression patterns were classified into six categories of inheritance depending on the significance of the differential expression measured by performing pairwise comparisons in all conditions, according to McManus et al.^13^. These categories are as follows: Conserved, when the level of gene expression in hybrids is similar to that of both parental lines (not show in the plot). Additive, when the expression levels are different between the two parental lines, but the hybrid expression level is intermediate. *D. arizonae*-dominant or *D. mojavensis*-dominant, when the hybrid expression level is similar to that of only one parental line. Overdominant, when the expression level in the hybrids is significantly higher than that in both parental lines. Underdominant, when the hybrid expression level is significantly lower than that in both parental lines. The bar plot was generated using the ggplot2 package (version 3.3.3) in R^79^.

We compared the level of gene expression in H♀ari♂m^moj^, H♀m^wri^♂ari and H♀ari♂m^wri^ with that in each parental line, following the six inheritance categories of McManus et al.^15^. Most of the genes in the ovaries and testes of the interspecific hybrids showed conserved expression (Fig. 7, Supplementary Tables S19 and S20). H♀ari♂m^moj^ showed conserved expression for 97.8% of the genes in ovaries and 78.5% of those in testes (Fig. 7, Supplementary Table S19). Few genes were classified as having additive expression in the female and male gonads (0.05 and 1.45%, respectively). In the dominant category, H♀ari♂m^moj^ exhibited an overrepresentation of genes with *D. arizonae*-like expression (1.8%, in ovaries and 9.5% in testes), and few genes were *D. mojavensis*-dominant (Fig. 7, Supplementary Table S19). In the overdominant and underdominant categories, almost no genes were found for ovaries (0.03% of DEGs). However, the testes showed several genes in these categories, reaching 0.5% for overdominant and 4.6% for underdominant genes (Fig. 7, Supplementary Table S19).

Similar to H♀ari♂m^moj^, most of the expressed genes in H♀m^wri^♂ari and H♀ari♂m^wri^ female and male gonads had conserved expression, but in testes, the level of conserved inheritance was lower than that in ovaries (Fig. 7, Supplementary Table S20). Additionally, very few genes in the ovaries (0.3 and 0.22%) and testes (1.1 and 1.7%) of H♀m^wri^♂ari and H♀ari♂m^wri^ displayed additive expression (Fig. 7, Supplementary Table S20), as was observed for H♀ari♂m^moj^. An interesting finding is that most of the genes classified in dominant categories displayed a *D. arizonae*-like pattern of expression in ovaries, but in testes, this pattern was related to the maternal line (Fig. 7, Supplementary Table S20). Considering the overdominant and underdominant categories, few genes were found for these categories in ovaries (0.16% in H♀m^wri^♂ari and 0.13% in H♀ari♂m^wri^) (Fig. 7, Supplementary Table S20). Similarly, in hybrid testes, very few genes were classified as over- (0.28% in H♀m^wri^♂ari and 0.5% in H♀ari♂m^wri^) or underdominant (0.12% in H♀m^wri^♂ari and 1.1% in H♀ari♂m^wri^) (Fig. 7, Supplementary Table S20).

Comparing all inheritance categories of H♀m^wri^♂ari and H♀ari♂m^wri^, significant differences in the expression profiles of reciprocal hybrids were observed in ovaries (*X*^2^ = 146.67, *p* < 0.001), which were mainly influenced by paternal effects, since 201 H♀m^wri^♂ari genes and 23 H♀ari♂m^wri^ genes showed paternally dominant inheritance. In testes, significant differences in the expression profiles were also found between reciprocal hybrids (*X*^2^ = 823.2, *p* < 0.001); however, unlike in the ovaries, these differences were mainly influenced by maternal and underdominant inheritance. More specifically, 140 and 695 genes were found to have exclusive maternal inheritance and 14 and 128 genes had underdominant expression in H♀m^wri^♂ari and H♀ari♂m^wri^, respectively.

## Discussion

In our study, the target species *D. m. mojavensis, D. m. wrigleyi* and *D. arizonae* showed approximately 6% of genes being differentially expressed in the ovaries and approximately 16% of genes being differentially expressed in the testes, agreeing with previous findings from our research group for hybrid female transcriptomes^30^ and with results obtained for hybrid male gonads^7^. This proportion of DEGs between species was intermediate when compared to other pairs of species presenting higher or lower divergence times. For example, *D. yakuba* and *D. melanogaster* (~ 6.1 mya divergence time^20^) have a proportion of DEGs varying from 29.59 to 42.58%^50^. In more recently diverged species, fewer DEGs are generally observed, such as in males of *D. yakuba* and *D. santomea* (~ 1.18 mya^51^, DEG: 19%^7^) and those of *D. p. pseudoobscura* and *D. p. bogotana* (~ 0.25 mya^52^, DEG: 14.6%^53^).

The differences in the proportion of DEGs in ovaries and testes could be related to the faster evolution of male-biased genes, as has been previously reported in other *Drosophila* species. According to previous studies, during the divergence process, male-biased genes display higher evolutionary rates, driven by positive selection, and most of them are preferentially expressed in gonad tissues^54–57^. This is the case in *D. arizonae* and *D. mojavensis* and can explain the high proportion of DEGs in testes when compared with the parental lines^58^.

Hybrid female gonads exhibited few DEGs when compared with both parental lines, and most of these genes were overexpressed. In agreement with our results, previous studies in hybrids of *D. arizonae-D. m. mojavensis*^30^ and *D. melanogaster-D. simulans*^13^ have reported a bias towards overexpression in female offspring. However, this bias was not observed in *D. buzzatii* and *D. koepferae* (~ 4.49 mya) female hybrids^14^. The absence of massive gene deregulation in ovaries could be related to the higher stability of gene expression in females, which is mainly influenced by the slower evolutionary rates of female-biased genes and the presence of two X-chromosomes, as was reported in other *Drosophila* species^17,57^, and this phenomenon could be important in the fertile phenotype observed in these hybrids. Thus, we suggest that in hybrid ovaries from recently diverged species, DEGs tend to be overexpressed, but over time, global deregulation will increase due to the accumulation of genetic changes, and the number of over- and underexpressed genes will become symmetric.

Male hybrids from crosses performed in different directions exhibited distinct proportions of genes with transgressive expression (over- or underexpressed relative to the parental species). In hybrids in which the mother was *D. m. wrigleyi*, we observed an overexpression of genes when compared to the parental lines. In contrast, if the mother was *D. arizonae*, there was either no difference between the proportion of over- or underexpressed genes or we observed a bias towards underexpression in relation to the parental lines. The differences observed between the reciprocal crosses could be due to the origin of the sex chromosomes. It has been reported that some specific epistatic factors among sex chromosomes and autosomes play a role in the differential expression profiles of interspecific hybrids^59^, which could explain the differences we observed.

Moreover, differences in sex-autosome interactions between reciprocal hybrids could also affect the severity of the sterility phenotype. There is evidence that in hybrids between *D. arizonae* females and *D. m. mojavensis* males, amotile sperm can result from interactions between the *D. m. mojavensis* Y chromosome and the 3rd autosome and/or the X chromosome from *D. arizonae*^60^. However, in the reciprocal cross, male hybrid sterility was associated with interactions between the Y chromosome of *D. arizonae* and the 4th autosome of *D. mojavensis*^61^. Our analyses of sperm motility and fertility showed that hybrid males from crosses between *D. arizonae* females and *D. m. mojavensis* or *D. m. wrigleyi* males had amotile sperm and were sterile, as expected. The sperm of hybrid males from crosses between *D. mojavensis* females and *D. arizonae* males was motile, exhibiting normal tails, but the individuals were sterile, indicating that the disruption of spermatogenesis also occurred in these males. These findings are in agreement with Hardy et al.^62^, who observed that *D. arizonae*-*D. m. mojavensis* hybrids display abnormal spermatid development and disruption of the spermatid tails.

The differential gene expression pattern in hybrid testes that we observed for crosses with *D. arizonae* mothers, with a tendency for an excess of underexpressed genes in hybrids when compared to the parental lines, is thus associated with a more severe male sterile phenotype. The functions associated with these underexpressed genes are related to spermatogenesis and could indicate a breakdown in gene regulation during spermatogenesis^55–57,63–65^. Moreover, we also identified genes related to metabolism, which supports the reduction of hybrid viability. These findings are in agreement with several studies that reported that most of the DEGs in male hybrids are underexpressed, and their functions are associated with reproduction, such as meiotic arrest, spermatogenesis^4,6,8^ and spermiogenesis, which are often observed in sterile hybrids^66–69^.

Analyses of the inheritance of gene expression patterns showed that most of the genes had conserved expression in hybrid ovaries and testes. This result is similar to previous findings for *D. arizonae-D. m. mojavensis* female hybrids^30^ but is quite different from those found in *D. sechellia*-*D. melanogaste*r and *D. simulans*-*D. melanogaster* hybrids, since only 6% and 11% of their genes had conserved expression, respectively^9,15^. Furthermore, given that *D. simulans*-*D. melanogaste*r and *D. arizonae*-*D. mojavensis* may have similar divergence times^21,70^, the number of genes classified as conserved is quite different (11% vs. ~ 95%), indicating that the interactions between different genetic changes accumulated in distinct species might have stronger or weaker effects on hybrids, causing disturbances in gene expression to variable degrees.

Among the DEGs with dominant inheritance in the ovaries and testes of all hybrids, most showed *D. arizonae*-like expression, regardless of the crossing direction. These findings could indicate that in the gonads of hybrids, the effect of the *D. arizonae* genome is more important than the maternal effects. Therefore, we speculate that for hybrid ovaries, the *D. arizonae* genome has a stronger effect on hybrid gene expression, likely due to regulatory sequences. This result corroborates our previous findings^30^ that among the genes classified in the dominant category, for both crossing directions, the *D. arizonae*-dominant inheritance pattern was stronger. Likewise, the expression profile in the ovaries of hybrids from crosses between *D. melanogaster* females and *D. sechellia* males showed that 49% of the genes were classified as dominant, and among these, 84% showed *D. sechellia*-like expression^15^, indicating that the maternal species does not influence the expression profile of the hybrids. In the target species of the current study, the hybrid female gonads showed very few genes classified as over- or underdominant, agreeing with previously reported results^30^ and demonstrating the greater stability of their gene expression. In testes, on the other hand, the number of differentially expressed genes classified in these categories was higher, with a higher number of underdominant genes for one specific cross direction. In hybrid male gonads, the underexpression of genes, mainly related to reproduction, has often been observed^15,56,71,72^, indicating that this can be related to disruptions in regulatory networks driven by the rapid divergence of male-biased genes, leading to sterility^7,56^.

Therefore, here, we showed that in hybrids from recently diverged species, such as *D. arizonae* and *D. mojavensis*, the degree of misregulated gene expression is related to the severity of the sterile phenotype. In female hybrids, which are fertile, very few DEGs were identified when compared with both parental lines. In contrast, in male hybrids, the degree of misregulated gene expression was dependent on the subspecies of *D. mojavensis* and, most importantly, the cross direction. Hybrid males from *D. mojavensis* females*,* which are sterile but had motile sperm, displayed a smaller number of misregulated genes, with a bias towards overexpression; these genes are not directly related to the sterile phenotype, since few genes acting on reproduction were found. Nevertheless, in male hybrids carrying the X chromosome of *D. arizonae,* which are sterile with amotile sperm*,* the disruption of gene expression was higher and presented a bias towards underexpression. Surprisingly, most of these genes were directly related to spermatogenesis-related functions, as well as sperm movement. However, more analyses must be undertaken to clarify the regulatory differences between this pair of species. These analyses include investigating the divergence of male-biased gene sequences, regulatory studies (cis–trans regulatory changes and the impact of microRNAs on gene expression), and functional analyses of the genes identified in the current study. Thus, we can obtain a better understanding of their relationship with the sterile phenotype in the initial steps of hybrid incompatibility.

## Methods

### *Drosophila* strains and RNA sequencing

Intraspecific and interspecific reciprocal crosses were performed between *D. arizonae* from Metztitlan, Hidalgo, México (stock number: 15081-1271.17) and two subspecies of *D. mojavensis*: *D. m. mojavensis* from the Anza Borrego Desert, California, USA (stock number: 15081-1352.01) and *D. m. wrigleyi* from Catalina Island, California, USA (stock number: 15081-1352.22). Crosses were performed with 3-day-old flies, ten males and ten females, in 2.3 × 9.5 cm vials containing standard *Drosophila* medium supplemented with yeast under the same temperature (23 °C) and humidity conditions. One-day-old virgin female and male offspring (control and F_1_ hybrids) were collected after hatching and were isolated until they reached sexual maturity. The male and female reproductive tracts of 9- to 12-day-old flies were dissected in PBS (phosphate-buffered saline) and stored at − 80 °C until use for RNA extraction.

To verify the hybrid status of the F_1_ offspring of interspecific crosses, 10 individuals of each cross were randomly collected for DNA extraction, and PCR for the ribosomal ITS-1 (*internal transcribed spacer 1*) from the 18S gene region (NCBI Reference Sequence: EU306666.1)^73^ was performed. The oligonucleotide primer for ITS-1 amplified 500 bp and 550 bp amplicons in *D. arizonae* and *D. mojavensis*, respectively. Therefore, in hybrids, two different fragments corresponding to *D. arizonae* and *D. mojavensis* alleles were expected.

After confirming the hybrid status of the offspring, 30 pairs of ovaries and 50 pairs of testes were used to perform total RNA extraction with two biological replicates using the RNeasy kit (Qiagen). The samples were treated with DNase (DNA-free Kit, Ambion) and stored at − 80 °C. The samples were quantified by fluorescence in a Bioanalyzer 2100 (Agilent). Sequencing was performed using the GenomEast platform by a member of the France Génomique consortium (ANR-10-INBS-0009) with an Illumina HiSeq 4000. The samples were sequenced in 2 × 100 paired-end reads, and the average size of the inserts was 300 base pairs.

Twelve transcriptomes were sequenced with two biological replicates each: *D. arizonae*, *D. m. mojavensis,* and *D. m. wrigleyi* (controls, ovaries and testes) and hybrids from crosses between *D. arizonae* and both *D. mojavensis* subspecies (ovaries and testes). The hybrid transcriptomes from *D. m. mojavensis* female and *D. arizonae* male crosses were not sequenced because the hybrid incompatibility in this direction was very high, and it was not possible to obtain enough material to perform RNA extraction. The low number of replicates is due to the high index of prezygotic reproductive isolation between *D. arizonae* and *D. mojavensis* subspecies (ranging from 0.56 to ~ 0.70^25^) and the low average viability of the hybrid offspring, which limited the number of hybrids obtained to perform RNA extraction.

### Mapping and quantification of expression

The sequenced transcriptomes were trimmed using UrQt^74^ to remove polyA tails and low-quality nucleotides. The sequence quality was then checked with FastQC software^75^. The transcriptomes were aligned against all annotated coding sequences (CDSs) of the *D. mojavensis* r1.04 public genome^76^ (available at http://flybase.org/). Overall, 21,915 Ref-Seq sequences were downloaded from https://www.ncbi.nlm.nih.gov/refseq/. From those sequences, 20,110 corresponding to mRNA were used as a reference to perform the alignments. This approach was used because the public genome of *D. mojavensis* presents the best quality of sequences and because *D. mojavensis* and *D. arizonae* are recently diverged species, a large divergence in their coding protein genes was not expected. Kallisto^77^ was used to map the reads from parental and hybrid transcriptomes against the *D. mojavensis* r1.04 reference transcripts. Kallisto is able to perform rapid pseudoalignment to quickly determine the compatibility of the reads with their respective targets. The pseudoalignment of reads preserves the key information needed for quantification and is robust against errors, presenting a similar accuracy as other alignment tools^77^. After the mapping procedure, BioMart^78^, an R (3.6.1)^79^ Bioconductor package, was used to recover the gene names corresponding to each transcript from the reference. This was possible because the BioMart database is maintained by Ensembl^80^, providing direct access to a diverse set of data and enabling a wide range of powerful online queries, from gene annotation to database mining. Subsequently, due to several genes displaying different isoforms, the package tximport was used to summarize the transcript level estimation for the gene level analysis, allowing us to use these data for the differential expression of gene-level counts.

### Differential expression analyses

Differential expression analyses were performed using DESeq2^81^, an R (3.6.1) package^79^, using raw read counts to identify differentially expressed genes in the hybrids compared to the parental species (controls lines) for each gonad tissue. This package normalizes the counts using size factors that are estimated according to the median counts taken for all genes. Additionally, DESeq2 estimates the means and variances of raw read counts and tests for differential expression based on a model using a negative binomial distribution and uses Benjamini–Hochberg multiple test correction (FDR level of 0.01). The low number of replicates can influence the statistical support of differential expression analyses. Therefore, to be conservative, we implemented stringent statistical thresholds. Genes were classified as significantly differentially expressed when the p-value, which was adjusted by FDR level, was below 0.01 and a higher than twofold change in expression was observed (corresponding to log2(FC) >|1|. Transcripts that presented fewer than ten mapped reads in all conditions tested were excluded from the analyses. The number of over- and underexpressed genes was analysed by a proportion test (prop.test) with R software^79^.

### Functional annotation and gene ontology enrichment analyses

Functional annotation was performed for all DEGs identified in the ovaries and testes of parents and hybrids. For this analysis, an orthologous gene table for *Drosophila* species was downloaded from http://flybase.org/. The *D. melanogaster* orthologs corresponding to DEGs in the hybrids were submitted to DAVID GO^82,83^. In addition, gene enrichment was investigated using a list of specific DEGs. Thus, a target gene list was compared with a reference list, which contained all the genes in the PANTHER Classification System platform^84–86^ (available at http://geneontology.org/) for a selected organism, using Fisher’s exact tests with FDR corrections or *p*-values. Then, we selected all significant GO terms from our target gene list and submitted them to the REVIGO web server^87^. By using REVIGO, we were able to summarize and remove redundant GO terms.

### Inheritance classifications

The R (3.6.1) package^79^ was used to sort genes in terms of differences in their expression levels between each parental line and the reciprocal hybrids separately, according to McManus et al.^15^. The expression data were transformed into log percentages, and a threshold of twofold change and adjusted *p*-value < 0.01 were set to determine the significance of differentially expressed genes. Genes that were not differentially expressed were considered to have the same expression level as the parental lines, thus being considered conserved. Genes considered differentially expressed were classified as additive, dominant, underdominant or overdominant. Additive expression means that the expression level of a given gene is different between the two parental lines but intermediate in the hybrid. *D. arizonae*-dominant or *D. mojavensis*-dominant expression is when the hybrid expression is similar to only one parental line. Overdominant expression means that expression in the hybrids is significantly higher than that in both parental lines, while underdominant expression means that the hybrid expression of a given gene is significantly lower than that in both parental lines. Chi-square statistical tests were performed in the R (3.6.1) package^79^.

### Viability and sterility analyses

Virgin males and females of each strain were separated by sex 10 h after eclosion and stored separately in yeasted cactus-banana vials, with 10 flies per vial, until flies were sexually mature (9 days of age). Crosses were performed between *D. m. mojavensis, D. m. wrigleyi* and *D. arizonae*, as well as within the parental lines, as control crosses. Five replicates were performed per cross with 10 couples in each vial, which favours mating^30^. Mating was performed for 72 h under similar temperature (23°) and light/dark (10/14 h) conditions. After 72 h, males from all crosses were discarded, and females (in pairs) were transferred to new fresh vials to lay eggs. This process was repeated five times every 48 h, so the females laid eggs for 10 days. Immediately after removing the females, the eggs that were laid were counted under a stereomicroscope, and after eclosion (~ 19 days after crossing), the number of imagoes was verified once a week for four weeks. The offspring viability (adults/eggs × 100) was calculated based on the number of eggs and adults.

For sterility analysis, interspecific and control crosses were performed using three-day-old virgin flies to obtain as many hybrids as possible. In previous tests, we noticed an increased production of hybrids when the two species were kept together before they reached sexual maturity. All crosses were performed with five replicates under the same temperature and light/dark conditions for 12 days. Then, the parents were discarded, and the imagoes were separated by sex daily. The offspring were maintained in yeasted food vials until they reached 10 days of age (sexually mature). Sperm motility analyses were carried out for 20 F_1_ male testes and seminal vesicles of each control and interspecific cross, according to Reed et al.^59^. No statistical analyses were performed because for each cross, all males presented the same phenotype, motile or amotile sperm. Additionally, fertility analyses were carried out by backcrossing female and male hybrids with their respective parents, *D. arizonae, D. m. mojavensis* and *D. m. wrigleyi*. Crosses were performed with five couples per replicate with five replicates by cross. To ensure that the absence of offspring was not due to possible prezygotic, post-mating-prezygotic or postzygotic isolation mechanisms, we increased the crossing time and allowed the couples to mate for 15 days. After that, all parents were discarded, and fertility was evaluated based on the presence or absence of offspring, as reported by Carnelossi et al.^28^. F_1_ × F_1_ crosses were also performed using the offspring of each interspecific cross under the same conditions as the backcrosses. To check that tubes containing only eggs would not produce offspring, the tubes were maintained for 20 days after parent removal and then discarded. Statistical analyses were performed for average fecundity by and viability for each replicate of intraspecific and interspecific crosses by using R (3.6.1) package^79^. Normality and variance tests (Shapiro–Wilk and Levene’s test, respectively) were carried out, and when we obtained significant *p*-values (non-normal distribution), a nonparametric Kruskal–Wallis test was performed. Then, a post hoc Wilcoxon test was performed to determine significant differences between the treatments. For results with no significant *p*-values for normality and variance tests, one-way ANOVA was performed using Tukey’s post hoc test.

## Supplementary Information

Supplementary Information.

Supplementary Tables.

## Data Availability

The datasets used and/or analysed during the current study are at https://www.ncbi.nlm.nih.gov/sra, Submission PRJNA691040.

## References

[1] Coyne JA, Orr HA (1997). Patterns of speciation in Drosophila revisited. Evolution.

[2] Orr HA, Coyne JA (1989). The genetics of postzygotic isolation in the Drosophila virilis group. Genetics.

[3] Turissini DA, McGirr JA, Patel SS, David JR, Matute DR (2018). The rate of evolution of postmating-prezygotic reproductive isolation in Drosophila. Mol. Biol. Evol..

[4] Brill E, Kang L, Michalak K, Michalak P, Price DK (2016). Hybrid sterility and evolution in Hawaiian Drosophila: differential gene and allele-specific expression analysis of bac.kcross males. Heredity (Edinb.).

[5] Hill T, Schlotterer C, Betancourt AJ (2016). Hybrid dysgenesis in Drosophila simulans associated with a rapid invasion of the P-element. PLoS Genet..

[6] Li R (2016). Specific down-regulation of spermatogenesis genes targeted by 22G RNAs in hybrid sterile males associated with an X-chromosome introgression. Genome Res..

[7] Llopart A (2012). The rapid evolution of X-linked male-biased gene expression and the large-X effect in *Drosophila yakuba*, *D. santomea*, and their hybrids. Mol. Biol. Evol..

[8] Moehring AJ, Teeter KC, Noor MA (2007). Genome-wide patterns of expression in Drosophila pure species and hybrid males. II. Examination of multiple-species hybridizations, platforms, and life cycle stages. Mol. Biol. Evol..

[9] Ranz JM, Namgyal K, Gibson G, Hartl DL (2004). Anomalies in the expression profile of interspecific hybrids of Drosophila melanogaster and Drosophila simulans. Genome Res..

[10] Ruiz A, Heed WB, Wasserman M (1990). Evolution of the mojavensis cluster of cactophilic Drosophila with descriptions of two new species. J. Hered..

[11] Dobzhansky T (1936). Studies on hybrid sterility. II. Localization of sterility factors in Drosophila Pseudoobscura hybrids. Genetics.

[12] Muller HJ (1942). Isolating mechanisms evolution and temperature. Biol Symp. 6,71–125 (1942). Biol. Symp..

[13] Kelleher ES, Edelman NB, Barbash DA (2012). Drosophila interspecific hybrids phenocopy piRNA-pathway mutants. PLoS Biol..

[14] Romero-Soriano V (2017). Transposable element misregulation is linked to the divergence between parental piRNA pathways in Drosophila hybrids. Genome Biol. Evol..

[15] McManus CJ (2010). Regulatory divergence in Drosophila revealed by mRNA-seq. Genome Res..

[16] Haldane JBS (1922). Sex ratio and unisexual sterility in animal hybrids. J. Genet..

[17] Llopart A, Brud E, Pettie N, Comeron JM (2018). Support for the dominance theory in drosophila transcriptomes. Genetics.

[18] Ryazansky, S., Mikhaleva, E., Akulenko, N. & Olenkina, O. Testis-expressed cluster of microRNAs 959–964 controls spermatid differentiation in *Drosophila*. 013243. 10.1101/013243 (2015).

[19] Reed LK, Nyboer M, Markow TA (2007). Evolutionary relationships of Drosophila mojavensis geographic host races and their sister species Drosophila arizonae. Mol. Ecol..

[20] Russo CA, Takezaki N, Nei M (1995). Molecular phylogeny and divergence times of drosophilid species. Mol. Biol. Evol..

[21] Sanchez-Flores A (2016). Genome evolution in three species of cactophilic Drosophila. G3 (Bethesda).

[22] Jennings JH, Etges WJ (2010). Species hybrids in the laboratory but not in nature: a reanalysis of premating isolation between *Drosophila arizonae* and *D. mojavensis*. Evolution.

[23] Etges WJ, De Oliveira CC, Noor MA, Ritchie MG (2010). Genetics of incipient speciation in Drosophila mojavensis. III. Life-history divergence in allopatry and reproductive isolation. Evolution.

[24] Machado CA, Matzkin LM, Reed LK, Markow TA (2007). Multilocus nuclear sequences reveal intra- and interspecific relationships among chromosomally polymorphic species of cactophilic Drosophila. Mol. Ecol..

[25] Massie KR, Markow TA (2005). Sympatry, allopatry and sexual isolation between *Drosophila mojavensis* and *D. arizonae*. Hereditas.

[26] Matzkin LM (2004). Population genetics and geographic variation of alcohol dehydrogenase (Adh) paralogs and glucose-6-phosphate dehydrogenase (G6pd) in Drosophila mojavensis. Mol. Biol. Evol..

[27] Matzkin LM (2008). The molecular basis of host adaptation in cactophilic Drosophila: molecular evolution of a glutathione S-transferase gene (GstD1) in *Drosophila mojavensis*. Genetics.

[28] Carnelossi EA (2014). Specific activation of an I-like element in Drosophila interspecific hybrids. Genome Biol. Evol..

[29] Reed LK, Markow TA (2004). Early events in speciation: polymorphism for hybrid male sterility in Drosophila. Proc. Natl. Acad. Sci. USA.

[30] Lopez-Maestre H (2017). Identification of misexpressed genetic elements in hybrids between Drosophila-related species. Sci. Rep..

[31] Dauwalder B, Tsujimoto S, Moss J, Mattox W (2002). The Drosophila takeout gene is regulated by the somatic sex-determination pathway and affects male courtship behavior. Genes Dev..

[32] Vanaphan N, Dauwalder B, Zufall RA (2012). Diversification of takeout, a male-biased gene family in Drosophila. Gene.

[33] Chatterjee N, Rollins J, Mahowald AP, Bazinet C (2011). Neurotransmitter Transporter-like: a male germline-specific SLC6 transporter required for Drosophila spermiogenesis. PLoS ONE.

[34] Courtot C, Fankhauser C, Simanis V, Lehner CF (1992). The Drosophila cdc25 homolog twine is required for meiosis. Development.

[35] Di Cara F, Cavaliere D, Galliero V, Polito LC, Digilio FA (2010). Expressional and functional analysis of the male-specific cluster mst36F during Drosophila spermatogenesis. Insect. Mol. Biol..

[36] Ellis LL, Carney GE (2010). Mating alters gene expression patterns in Drosophila melanogaster male heads. BMC Genom..

[37] Fabrizio JJ (2012). Mulet (mlt) encodes a tubulin-binding cofactor E-like homolog required for spermatid individualization in Drosophila melanogaster. Fly (Austin).

[38] Gumy LF (2013). The kinesin-2 family member KIF3C regulates microtubule dynamics and is required for axon growth and regeneration. J. Neurosci..

[39] Henson JH (1997). The heterotrimeric motor protein kinesin-II localizes to the midpiece and flagellum of sea urchin and sand dollar sperm. Cell Motil. Cytoskeleton.

[40] Ito S (2012). Epigenetic silencing of core histone genes by HERS in Drosophila. Mol. Cell.

[41] Karak S (2015). Diverse roles of axonemal dyneins in Drosophila auditory neuron function and mechanical amplification in hearing. Sci. Rep..

[42] Lin CJ (2017). Characterization of a TUTase/RNase complex required for Drosophila gametogenesis. RNA.

[43] Lin TY (1996). Coordinate developmental control of the meiotic cell cycle and spermatid differentiation in Drosophila males. Development.

[44] Rogers SL, Rogers GC, Sharp DJ, Vale RD (2002). Drosophila EB1 is important for proper assembly, dynamics, and positioning of the mitotic spindle. J. Cell Biol..

[45] Schafer M, Borsch D, Hulster A, Schafer U (1993). Expression of a gene duplication encoding conserved sperm tail proteins is translationally regulated in Drosophila melanogaster. Mol. Cell Biol..

[46] Szafer-Glusman E (2008). A role for very-long-chain fatty acids in furrow ingression during cytokinesis in Drosophila spermatocytes. Curr. Biol..

[47] Wijesekera TP, Saurabh S, Dauwalder B (2016). Juvenile hormone is required in adult males for Drosophila courtship. PLoS ONE.

[48] Zhang J, Luo J, Chen J, Dai J, Montell C (2020). The role of Y chromosome genes in male fertility in Drosophila melanogaster. Genetics.

[49] Zur Lage P, Newton FG, Jarman AP (2019). Survey of the ciliary motility machinery of Drosophila sperm and ciliated mechanosensory neurons reveals unexpected cell-type specific variations: a model for motile ciliopathies. Front. Genet..

[50] Rifkin SA, Kim J, White KP (2003). Evolution of gene expression in the Drosophila melanogaster subgroup. Nat. Genet..

[51] Turissini DA, Matute DR (2017). Fine scale mapping of genomic introgressions within the Drosophila yakuba clade. PLoS Genet..

[52] Wang RL, Wakeley J, Hey J (1997). Gene flow and natural selection in the origin of Drosophila pseudoobscura and close relatives. Genetics.

[53] Gomes S, Civetta A (2015). Hybrid male sterility and genome-wide misexpression of male reproductive proteases. Sci. Rep..

[54] Artieri CG, Haerty W, Singh RS (2007). Association between levels of coding sequence divergence and gene misregulation in Drosophila male hybrids. J. Mol. Evol..

[55] Civetta A, Rajakumar SA, Brouwers B, Bacik JP (2006). Rapid evolution and gene-specific patterns of selection for three genes of spermatogenesis in Drosophila. Mol. Biol. Evol..

[56] Haerty W, Singh RS (2006). Gene regulation divergence is a major contributor to the evolution of Dobzhansky–Muller incompatibilities between species of Drosophila. Mol. Biol. Evol..

[57] Meiklejohn CD, Parsch J, Ranz JM, Hartl DL (2003). Rapid evolution of male-biased gene expression in Drosophila. Proc. Natl. Acad. Sci. USA.

[58] Bono JM, Matzkin LM, Hoang K, Brandsmeier L (2015). Molecular evolution of candidate genes involved in post-mating-prezygotic reproductive isolation. J. Evol. Biol..

[59] Reed LK, LaFlamme BA, Markow TA (2008). Genetic architecture of hybrid male sterility in Drosophila: analysis of intraspecies variation for interspecies isolation. PLoS ONE.

[60] Vigneault G, Zouros E (1986). The genetics of asymmetrical male sterility in *Drosophila mojavensis* and Drosophila Arizonensis hybrids: interactions between the Y-chromosome and autosomes. Evolution.

[61] Pantazidis AC, Zouros E (1988). Location of an autosomal factor causing sterility in Drosophila mojavensis males carrying the Drosophila arizonensis Y chromosome. Heredity (Edinb.).

[62] Hardy RW, Lougheed A, Markow TA (2011). Reproductive tract and spermatid abnormalities of hybrid males from reciprocal crosses between *Drosophila mojavensis* and *D. arizonae*. Fly (Austin).

[63] Sundararajan V, Civetta A (2011). Male sex interspecies divergence and down regulation of expression of spermatogenesis genes in Drosophila sterile hybrids. J. Mol. Evol..

[64] Wu CI, Davis AW (1993). Evolution of postmating reproductive isolation: the composite nature of Haldane's rule and its genetic bases. Am. Nat..

[65] Bauer DuMont VL, Flores HA, Wright MH, Aquadro CF (2007). Recurrent positive selection at bgcn, a key determinant of germ line differentiation, does not appear to be driven by simple coevolution with its partner protein bam. Mol. Biol. Evol..

[66] Jiang J, White-Cooper H (2003). Transcriptional activation in Drosophila spermatogenesis involves the mutually dependent function of aly and a novel meiotic arrest gene cookie monster. Development.

[67] Lindsley DL, Roote J, Kennison JA (2013). Anent the genomics of spermatogenesis in Drosophila melanogaster. PLoS ONE.

[68] Moehring AJ, Llopart A, Elwyn S, Coyne JA, Mackay TF (2006). The genetic basis of postzygotic reproductive isolation between *Drosophila santomea* and *D. yakuba* due to hybrid male sterility. Genetics.

[69] White-Cooper H, Schafer MA, Alphey LS, Fuller MT (1998). Transcriptional and post-transcriptional control mechanisms coordinate the onset of spermatid differentiation with meiosis I in Drosophila. Development.

[70] Cutter AD (2008). Divergence times in Caenorhabditis and Drosophila inferred from direct estimates of the neutral mutation rate. Mol. Biol. Evol..

[71] Gomes S, Civetta A (2014). Misregulation of spermatogenesis genes in Drosophila hybrids is lineage-specific and driven by the combined effects of sterility and fast male regulatory divergence. J. Evol. Biol..

[72] Ometto L, Ross KG, Shoemaker D, Keller L (2012). Disruption of gene expression in hybrids of the fire ants Solenopsis invicta and *Solenopsis richteri*. Mol. Ecol..

[73] Baffi MA, Ceron CR (2002). Molecular analysis of the rDNA ITS-1 intergenic spacer in *Drosophila mulleri*, *D. arizonae*, and their hybrids. Biochem. Genet..

[74] Modolo L, Lerat E (2015). UrQt: an efficient software for the unsupervised quality trimming of NGS data. BMC Bioinform..

[75] S., A. *FastQC: a quality control tool for high throughput sequence data*. http://www.bioinformatics.babraham.ac.uk/projects/fastqc (2010).

[76] Drosophila 12 Genomes, C. *et al.* Evolution of genes and genomes on the Drosophila phylogeny. *Nature* **450**, 203–218. 10.1038/nature06341 (2007). 10.1038/nature06341 17994087

[77] Bray NL, Pimentel H, Melsted P, Pachter L (2016). Near-optimal probabilistic RNA-seq quantification. Nat. Biotechnol..

[78] Durinck S (2005). BioMart and Bioconductor: a powerful link between biological databases and microarray data analysis. Bioinformatics.

[79] Team, R. C. R: A language and environment for statistical computing. *R Foundation for Statistical Computing, Vienna, Austria. *https://www.R-project.org/*.* (2018).

[80] Yates AD (2020). Ensembl 2020. Nucl. Acids Res..

[81] Love MI, Huber W, Anders S (2014). Moderated estimation of fold change and dispersion for RNA-seq data with DESeq2. Genome Biol..

[82] da Huang W, Sherman BT, Lempicki RA (2009). Systematic and integrative analysis of large gene lists using DAVID bioinformatics resources. Nat. Protoc..

[83] da Huang W, Sherman BT, Lempicki RA (2009). Bioinformatics enrichment tools: paths toward the comprehensive functional analysis of large gene lists. Nucl. Acids Res..

[84] Ashburner M (2000). Gene ontology: tool for the unification of biology. The Gene Ontology Consortium. Nat. Genet..

[85] Mi H, Muruganujan A, Thomas PD (2013). PANTHER in 2013: modeling the evolution of gene function, and other gene attributes, in the context of phylogenetic trees. Nucl. Acids Res..

[86] Thomas PD (2003). PANTHER: a library of protein families and subfamilies indexed by function. Genome Res..

[87] Supek F, Bosnjak M, Skunca N, Smuc T (2011). REVIGO summarizes and visualizes long lists of gene ontology terms. PLoS ONE.

