# Supplementary material for: Comparative transcriptomics between Drosophila mojavensis and D. arizonae reveals transgressive gene expression and underexpression of spermatogenesis-related genes in hybrid testes
Source: Sci Rep. 2021 May 10;11:9844. doi: 10.1038/s41598-021-89366-2 (PMC8110761; doi:10.1038/s41598-021-89366-2)
Supplement: Supplementary file 1 — Supplementary Information. [file 41598_2021_89366_MOESM1_ESM.docx]

**Comparative transcriptomics between *Drosophila mojavensis* and *D. arizonae* reveals transgressive gene expression and underexpression of spermatogenesis-related genes in hybrid testes**

Cecilia A. Banho,^1,2^ Vincent Mérel,^2^ Thiago Y. K. Oliveira,^3^ Claudia M. A. Carareto,^1^ and Cristina Vieira^2*^

**Supplementary material**


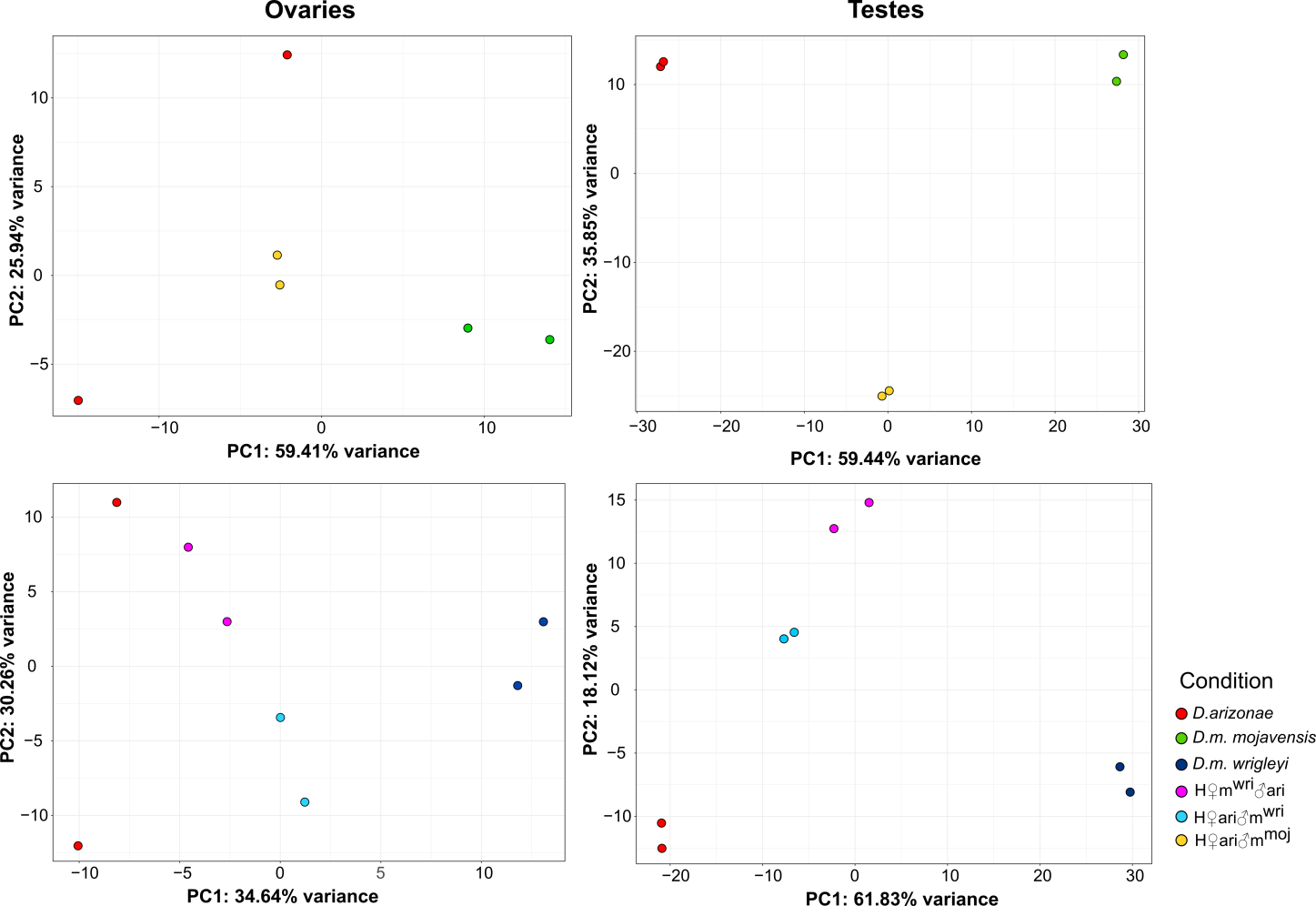


**Supplementary Fig. S1. Principal component analysis (PCA)**. Transcriptome replicates of ovaries (on the left) and testes (on the right) for *D. arizonae, D. m. mojavensis, D. m. wrigleyi*, H♀m^wri^♂ari, H♀ari♂m^wri^ and H♀ari♂m^moj^. The plot was generated using the ggplot2 package (version 3.3.3) in R^79^.


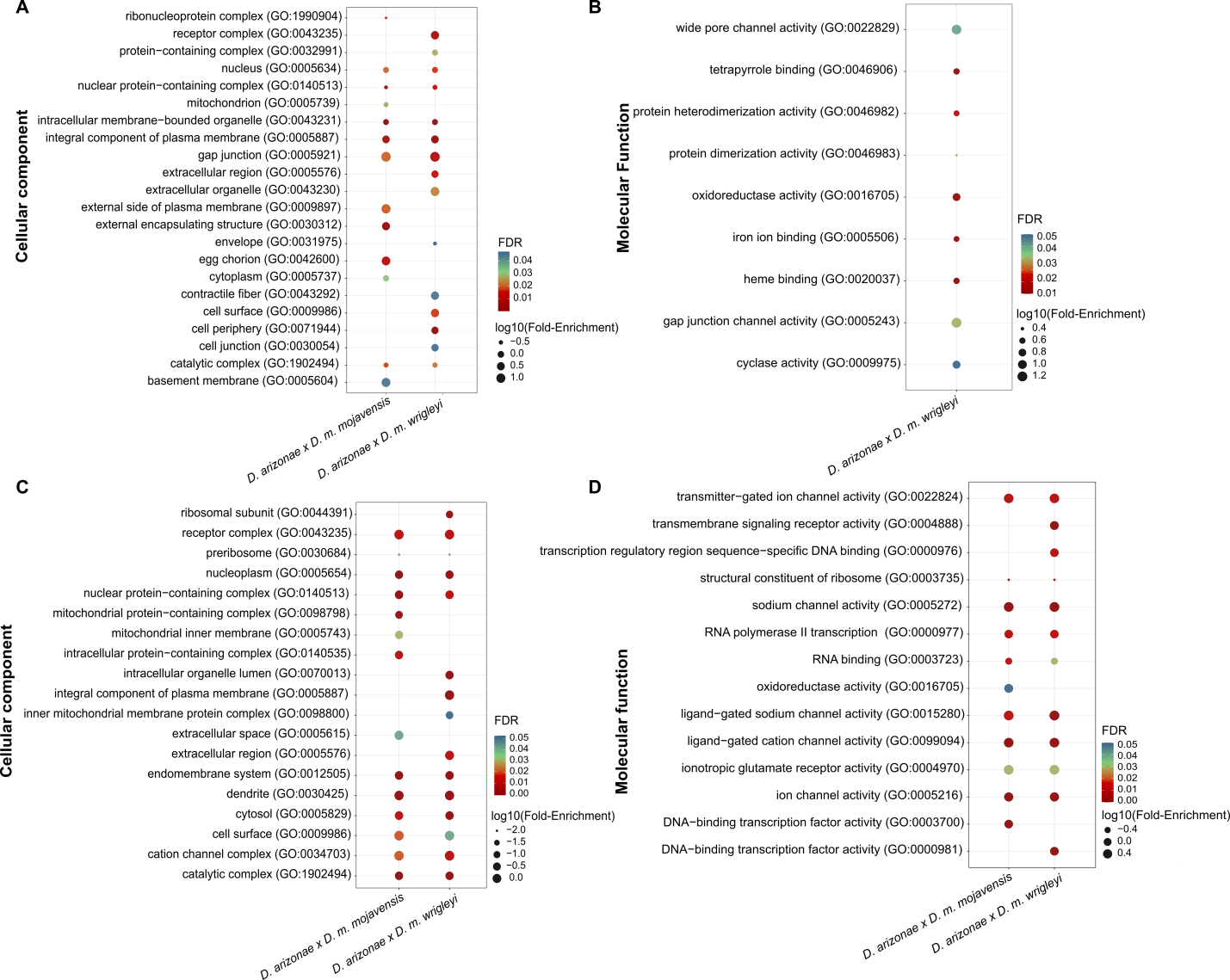


**Supplementary Figure S2. GO term enrichment analysis of DEGs between *D. arizonae* and *D. mojavensis* subspecies.** Dot plot representing GO enrichment for cellular component and molecular function for DEGs in A, B) ovaries and C, D) testes between *D. arizonae*- *D. m. mojavensis* and *D. arizonae-D. m. wrigleyi* (FDR < 0.05). The dot plot was generated using the ggplot2 package (version 3.3.3) in R^79^.
